# Supplementary material for: Dissection of core promoter syntax through single nucleotide resolution modeling of transcription initiation
Source: bioRxiv. 2024 Sep 17:2024.03.13.583868. Preprint. [Version 4] doi: 10.1101/2024.03.13.583868 (PMC10979970; doi:10.1101/2024.03.13.583868)
Supplement: 11 [file NIHPP2024.03.13.583868v4-supplement-11.pdf]

bioRxiv preprint doi: https://doi.org/10.1101/2024.03.13.583868; this version posted September 17, 2024. The copyright holder for this preprint (which was not certified by peer review) is the author/funder, who has granted bioRxiv a license to display the preprint in perpetuity. It is made available under aCC-BY 4.0 International license.

Supplementary Figures

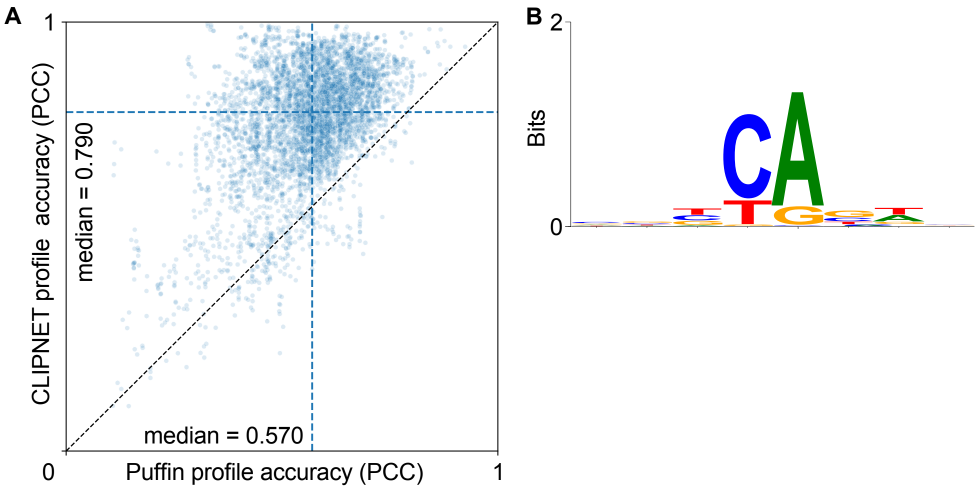

Fig. S1 Additional evaluation metrics for CLIPNET. (**A**) Scatterplot of CLIPNET and Puffin profile prediction accuracy. (**B**) Sequence logo of the predicted TSS motif from CLIPNET.

bioRxiv preprint doi: https://doi.org/10.1101/2024.03.13.583868; this version posted September 17, 2024. The copyright holder for this preprint (which was not certified by peer review) is the author/funder, who has granted bioRxiv a license to display the preprint in perpetuity. It is made available under aCC-BY 4.0 International license.

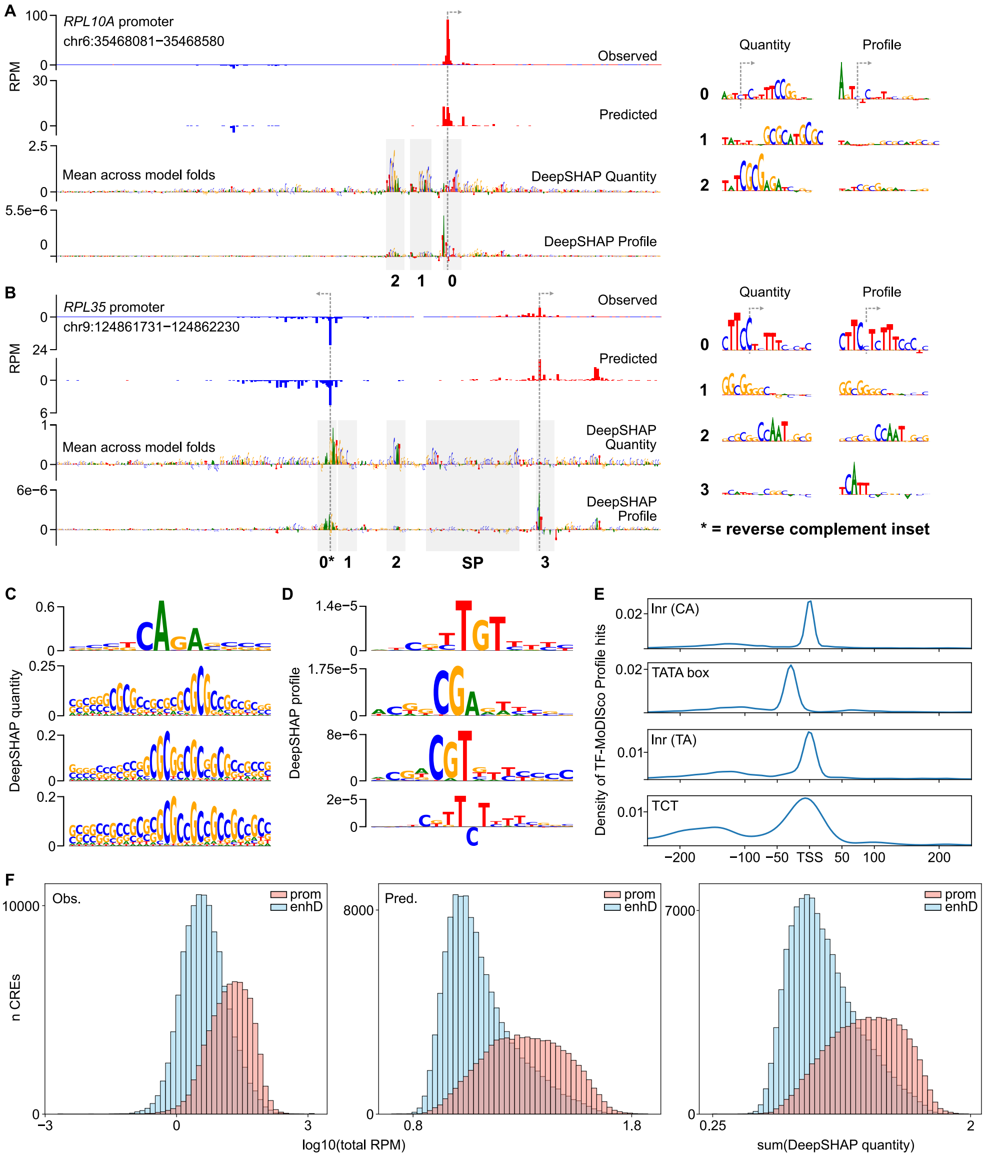

Fig. S2 Additional interpretation of CLIPNET with DeepSHAP and TF-MoDISco. (**A**, **B**) Prediction and DeepSHAP quantity and profile scores for the promoters of the ribosomal protein coding genes *RPL10A* (**A**) and *RPL35* (**B**). Both promoters use a TCT box instead of the canonical CA or TA initiators, which is correctly recognized by CLIPNET. Interesting motifs are highlighted in insets to the right. (**C**) Promoters have much higher total initiation than distal enhancers do (experiment, left; predicted, middle), which is reflected in the number and strength of individual motifs (DeepSHAP quantity, right; Fig. 2C). (**C**) Initiator and TATA box motifs identified by TF-MoDISco quantity. (**D**) Three examples of CpG-rich motifs identified by TF-MoDISco quantity. (**E**) Three non-canonical initiators identified by TF-MoDISco profile. (**F**) Distribution of observed (left) and predicted (center) transcription quantity and DeepSHAP quantity scores (right) at promoters and distal enhancers.

bioRxiv preprint doi: https://doi.org/10.1101/2024.03.13.583868; this version posted September 17, 2024. The copyright holder for this preprint (which was not certified by peer review) is the author/funder, who has granted bioRxiv a license to display the preprint in perpetuity. It is made available under aCC-BY 4.0 International license.

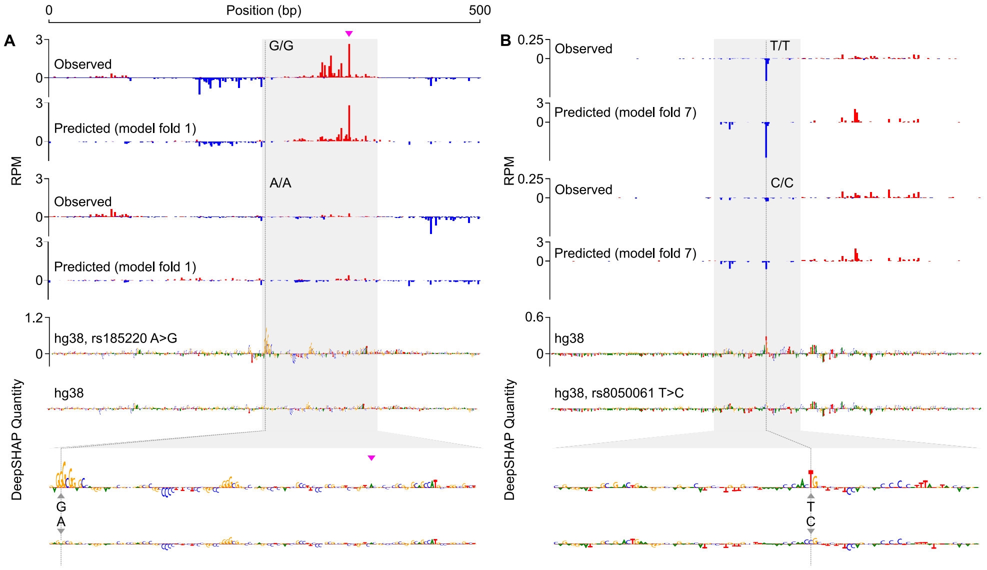

Fig. S3 DeepSHAP profile interpretation of QTL effects. (**A**, **B**) Same as Fig. 3C, D, but showing the DeepSHAP quantity scores for each variant.

bioRxiv preprint doi: https://doi.org/10.1101/2024.03.13.583868; this version posted September 17, 2024. The copyright holder for this preprint (which was not certified by peer review) is the author/funder, who has granted bioRxiv a license to display the preprint in perpetuity. It is made available under aCC-BY 4.0 International license.

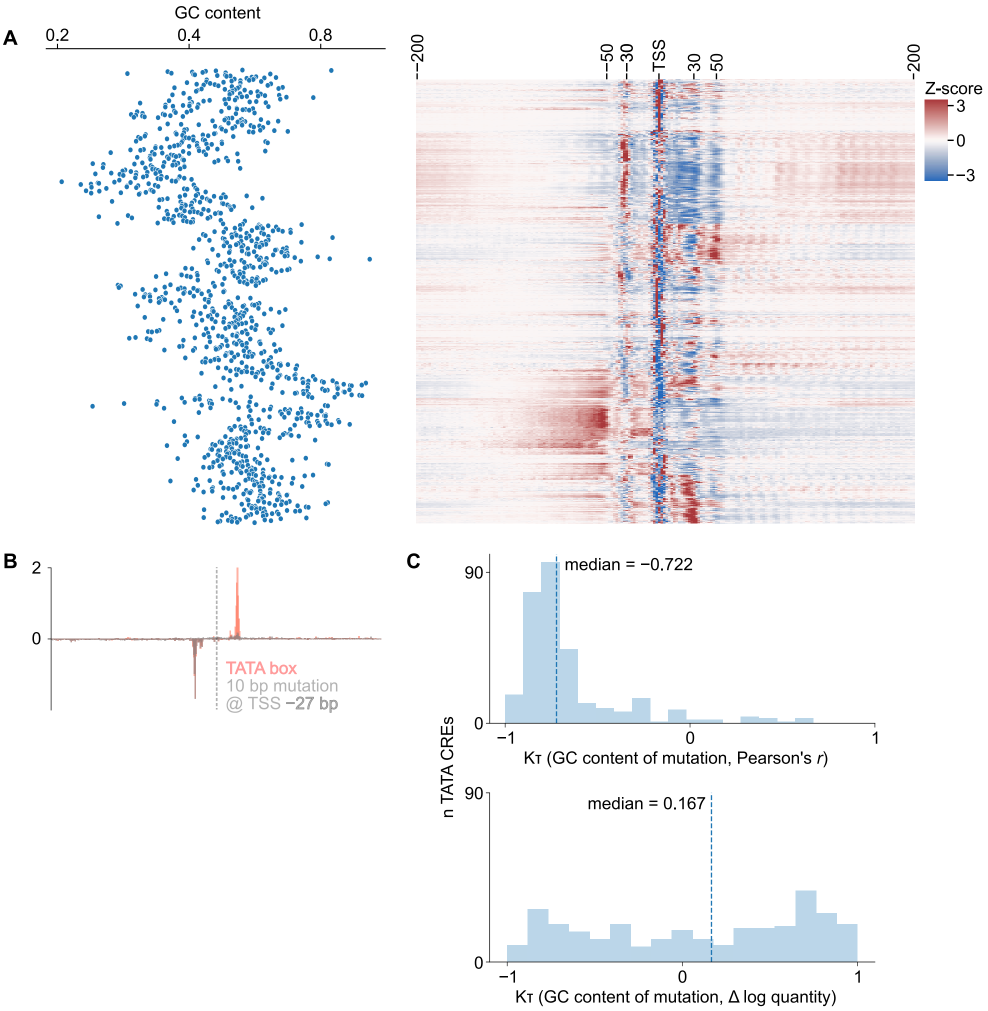

Fig. S4 Additional evaluation metrics for CLIPNET (**A**) Positional distribution of filter activations in the second convolutional layer (receptive field = 15, right) and the GC content of sequences driving maximal activation for these filters (left). (**B**) Metaplot of motif-directed mutagenesis of canonical TATA box motifs. (**C**) Monotonicity of the effect of GC shift mutagenesis of TATA boxes on initiation profile (top) and quantity (bottom).
