# Supplementary material for: Dissection of core promoter syntax through single nucleotide resolution modeling of transcription initiation
Source: bioRxiv. 2024 Apr 3:2024.03.13.583868. Preprint. [Version 3] doi: 10.1101/2024.03.13.583868 (PMC10979970; doi:10.1101/2024.03.13.583868)
Supplement: Supplementary file 1 [file NIHPP2024.03.13.583868V3-supplement-1.pdf]

bioRxiv preprint doi: https://doi.org/10.1101/2024.03.13.583868; this version posted April 3, 2024. The copyright holder for this preprint (which was not certified by peer review) is the author/funder, who has granted bioRxiv a license to display the preprint in perpetuity. It is made available under aCC-BY 4.0 International license.

Supplementary Figures

A

Model folds

	median_track_pcc	tss_pcc	log_quantity_pcc	test_fold	test_chrom	val_fold	val_chrom
1	0.674	0.759	0.598	1	5,7	2	12,11
2	0.677	0.757	0.608	2	12,11	3	14,15,18
3	0.664	0.743	0.580	3	14,15,18	4	1
4	0.680	0.768	0.604	4	1	5	2,22
5	0.659	0.722	0.589	5	2,22	6	4,6
6	0.657	0.732	0.532	6	4,6	7	3,16
7	0.644	0.743	0.564	7	3,16	8	19,10
8	0.650	0.712	0.591	8	19,10	9	17,8
9	0.670	0.726	0.547	9	17,8	1	5,7

B

Model folds

	median_track_pcc	tss_pcc	log_quantity_pcc	test_fold	test_chrom	val_fold	val_chrom
1	0.686	0.760	0.587	1	5,7	2	12,11
2	0.689	0.760	0.592	2	12,11	3	14,15,18
3	0.657	0.705	0.585	3	14,15,18	4	1
4	0.670	0.745	0.576	4	1	5	2,22
5	0.653	0.736	0.573	5	2,22	6	4,6
6	0.666	0.710	0.567	6	4,6	7	3,16
7	0.633	0.744	0.551	7	3,16	8	19,10
8	0.644	0.705	0.475	8	19,10	9	17,8
9	0.659	0.751	0.492	9	17,8	1	5,7

C

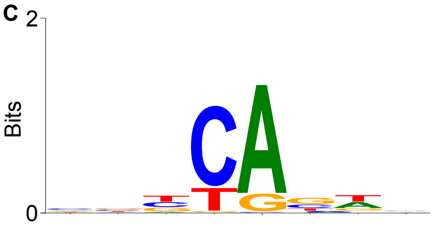

Fig. S1 Additional evaluation metrics for CLIPNET. (**A**, **B**) Performance metrics for the individual model folds when evaluated on the fold 0 (**A**) or on the individual holdout folds for each model (**B**). (**C**) Sequence logo of the predicted TSS motif.

bioRxiv preprint doi: https://doi.org/10.1101/2024.03.13.583868; this version posted April 3, 2024. The copyright holder for this preprint (which was not certified by peer review) is the author/funder, who has granted bioRxiv a license to display the preprint in perpetuity. It is made available under aCC-BY 4.0 International license.

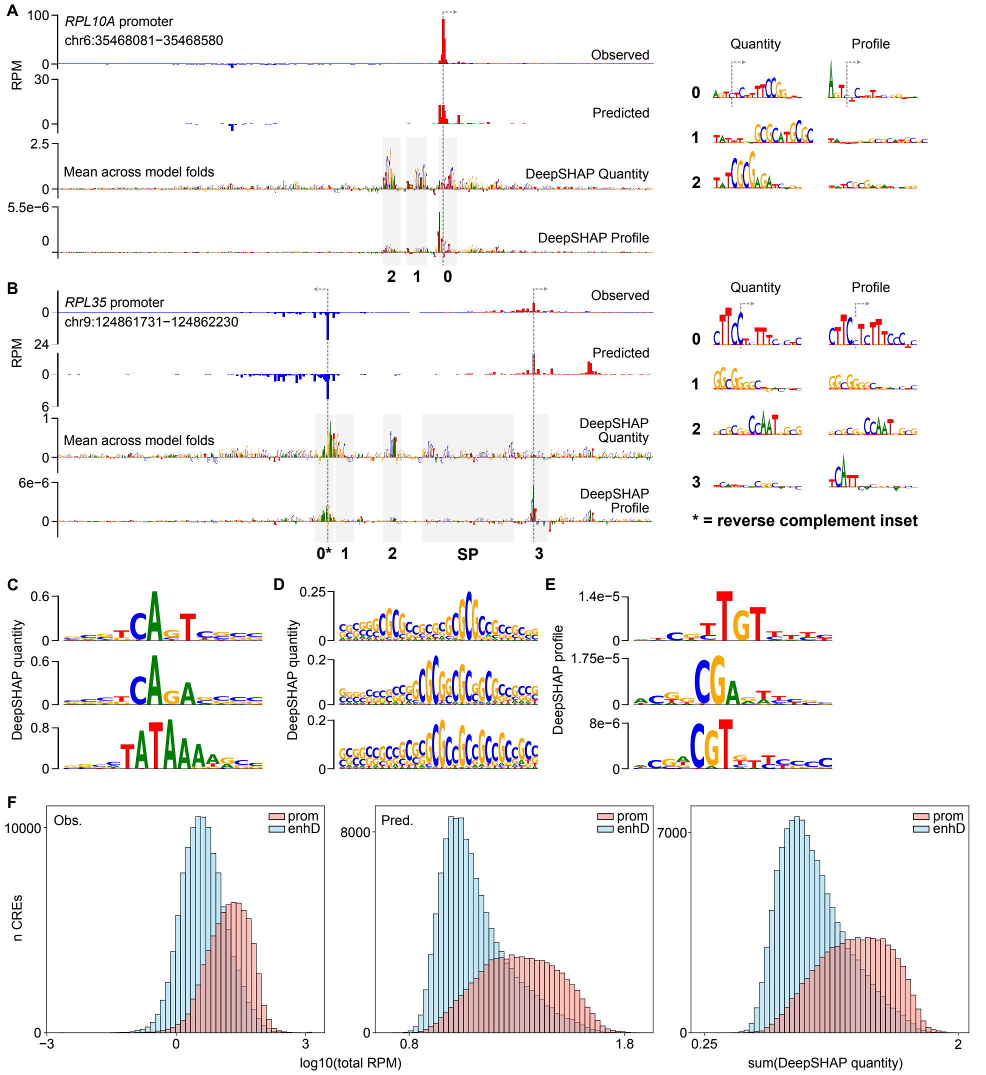

Fig. S2 Additional interpretation of CLIPNET with DeepSHAP and TF-MoDISco. (**A**, **B**) Prediction and DeepSHAP quantity and profile scores for the promoters of the ribosomal protein coding genes *RPL10A* (**A**) and *RPL35* (**B**). Both promoters use a TCT box instead of the canonical CA or TA initiators, which is correctly recognized by CLIPNET. Interesting motifs are highlighted in insets to the right. (**C**) Promoters have much higher total initiation than distal enhancers do (experiment, left; predicted, middle), which is reflected in the number and strength of individual motifs (DeepSHAP quantity, right; Fig. 2C). (**C**) Initiator and TATA box motifs identified by TF-MoDISco quantity. (**D**) Three examples of CpG-rich motifs identified by TF-MoDISco quantity. (**E**) Three non-canonical initiators identified by TF-MoDISco profile. (**F**) Distribution of observed (left) and predicted (center) transcription quantity and DeepSHAP quantity scores (right) at promoters and distal enhancers.

bioRxiv preprint doi: https://doi.org/10.1101/2024.03.13.583868; this version posted April 3, 2024. The copyright holder for this preprint (which was not certified by peer review) is the author/funder, who has granted bioRxiv a license to display the preprint in perpetuity. It is made available under aCC-BY 4.0 International license.

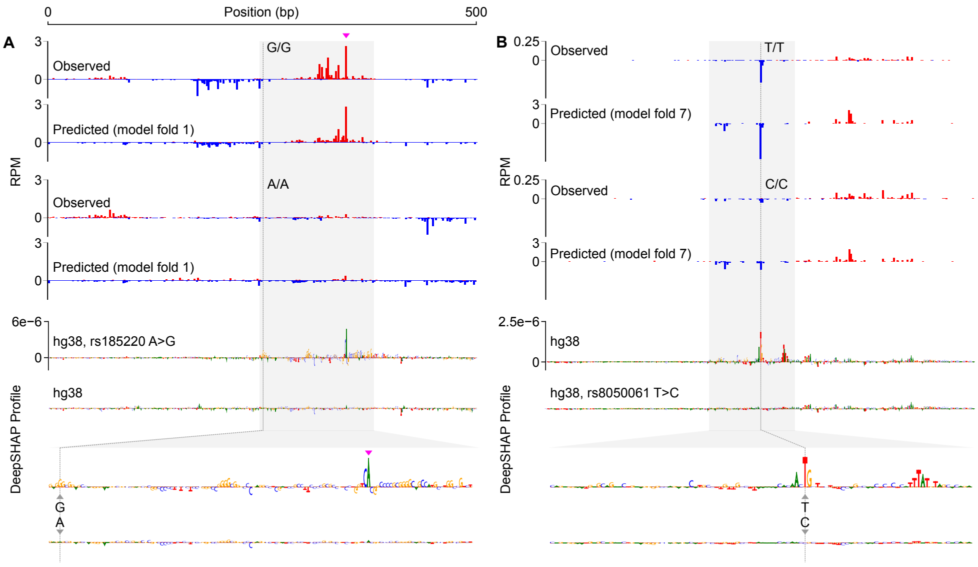

Fig. S3 DeepSHAP profile interpretation of QTL effects. (**A**, **B**) Same as Fig. 3C, D, but showing the DeepSHAP profile scores for each variant.

bioRxiv preprint doi: https://doi.org/10.1101/2024.03.13.583868; this version posted April 3, 2024. The copyright holder for this preprint (which was not certified by peer review) is the author/funder, who has granted bioRxiv a license to display the preprint in perpetuity. It is made available under aCC-BY 4.0 International license.

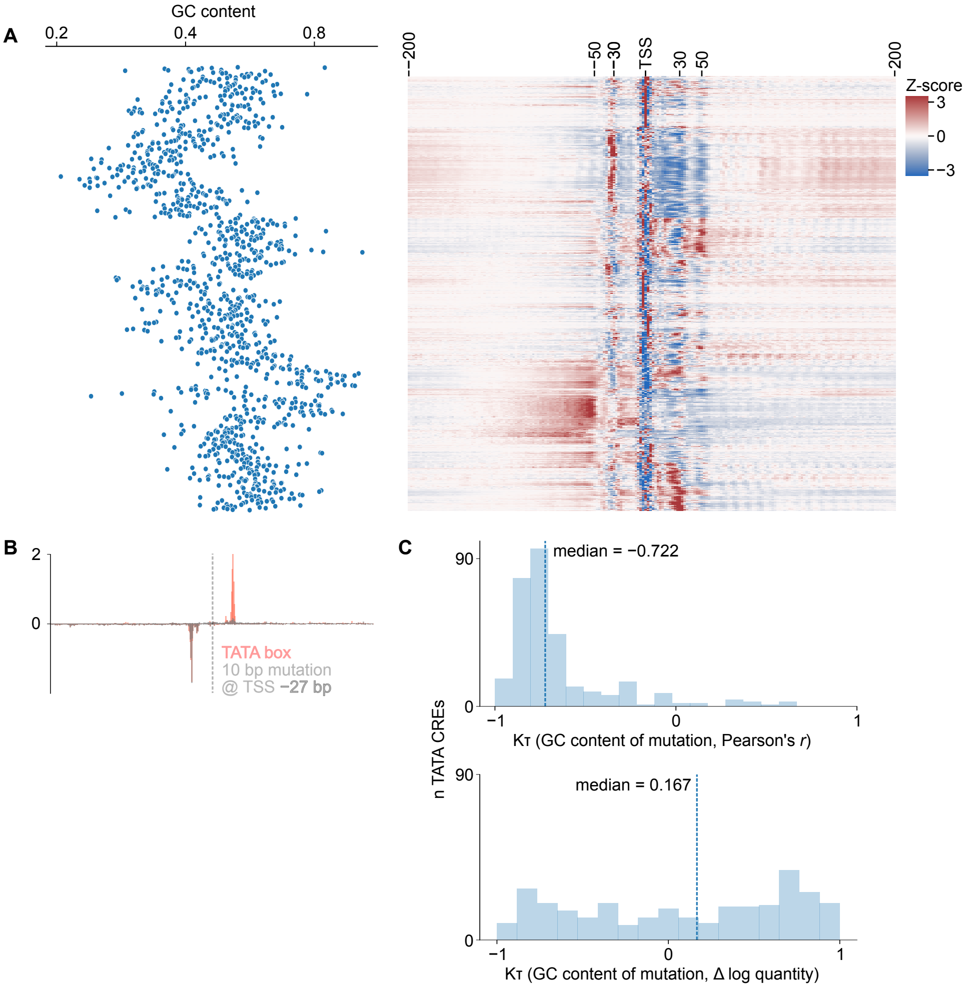

Fig. S4 Additional evaluation metrics for CLIPNET (**A**) Positional distribution of filter activations in the second convolutional layer (receptive field = 15, right) and the GC content of sequences driving maximal activation for these filters (left). (**B**) Metaplot of motif-directed mutagenesis of canonical TATA box motifs. (**C**) Monotonicity of the effect of GC shift mutagenesis of TATA boxes on initiation profile (top) and quantity (bottom).

Supplementary Information

Below are a brief description of each Supplementary Information file. Files are available online at https://doi.org/10.1101/2024.03.13.583868.

1

List of GEO accession IDs used to train CLIPNET (txt file).

bioRxiv preprint doi: https://doi.org/10.1101/2024.03.13.583868; this version posted April 3, 2024. The copyright holder for this preprint (which was not certified by peer review) is the author/funder, who has granted bioRxiv a license to display the preprint in perpetuity. It is made available under aCC-BY 4.0 International license.

2

High confidence PRO-cap peaks used to evaluate CLIPNET (bed file).

3

TF-MoDISco report for DeepSHAP quantity interpretation of CLIPNET (zip archive).

4

TF-MoDISco report for DeepSHAP profile interpretation of CLIPNET (zip archive).

5

tiQTLs used to benchmark CLIPNET (txt file, rsIDs).

6

diQTLs used to benchmark CLIPNET (txt file, rsIDs).

7

A random subset ($n = 5000$) of Supplementary Info. 2 (bed file).

8

Active TATA-containing *cis*-regulatory elements (bed file).

9

Active IRF4-containing *cis*-regulatory elements (bed file).

10

Active SP1-containing *cis*-regulatory elements (bed file).
