# Supplementary material for: Dissection of core promoter syntax through single nucleotide resolution modeling of transcription initiation
Source: bioRxiv. 2024 Mar 16:2024.03.13.583868. Preprint. [Version 2] doi: 10.1101/2024.03.13.583868 (PMC10979970; doi:10.1101/2024.03.13.583868)

Supplementary Figure 1

A

Model folds

	median_track_pcc	tss_pcc	log_quantity_pcc	test_fold	test_chrom	val_fold	val_chrom
1	0.674	0.759	0.598	1	5,7	2	12,11
2	0.677	0.757	0.608	2	12,11	3	14,15,18
3	0.664	0.743	0.580	3	14,15,18	4	1
4	0.680	0.768	0.604	4	1	5	2,22
5	0.659	0.722	0.589	5	2,22	6	4,6
6	0.657	0.732	0.532	6	4,6	7	3,16
7	0.644	0.743	0.564	7	3,16	8	19,10
8	0.650	0.712	0.591	8	19,10	9	17,8
9	0.670	0.726	0.547	9	17,8	1	5,7

B

Model folds

	median_track_pcc	tss_pcc	log_quantity_pcc	test_fold	test_chrom	val_fold	val_chrom
1	0.686	0.760	0.587	1	5,7	2	12,11
2	0.689	0.760	0.592	2	12,11	3	14,15,18
3	0.657	0.705	0.585	3	14,15,18	4	1
4	0.670	0.745	0.576	4	1	5	2,22
5	0.653	0.736	0.573	5	2,22	6	4,6
6	0.666	0.710	0.567	6	4,6	7	3,16
7	0.633	0.744	0.551	7	3,16	8	19,10
8	0.644	0.705	0.475	8	19,10	9	17,8
9	0.659	0.751	0.492	9	17,8	1	5,7

bioRxiv preprint doi: https://doi.org/10.1101/2024.03.13.583868; this version posted March 16, 2024. The copyright holder for this preprint (which was not certified by peer review) is the author/funder, who has granted bioRxiv a license to display the preprint in perpetuity. It is made available under aCC-BY 4.0 International license.

C

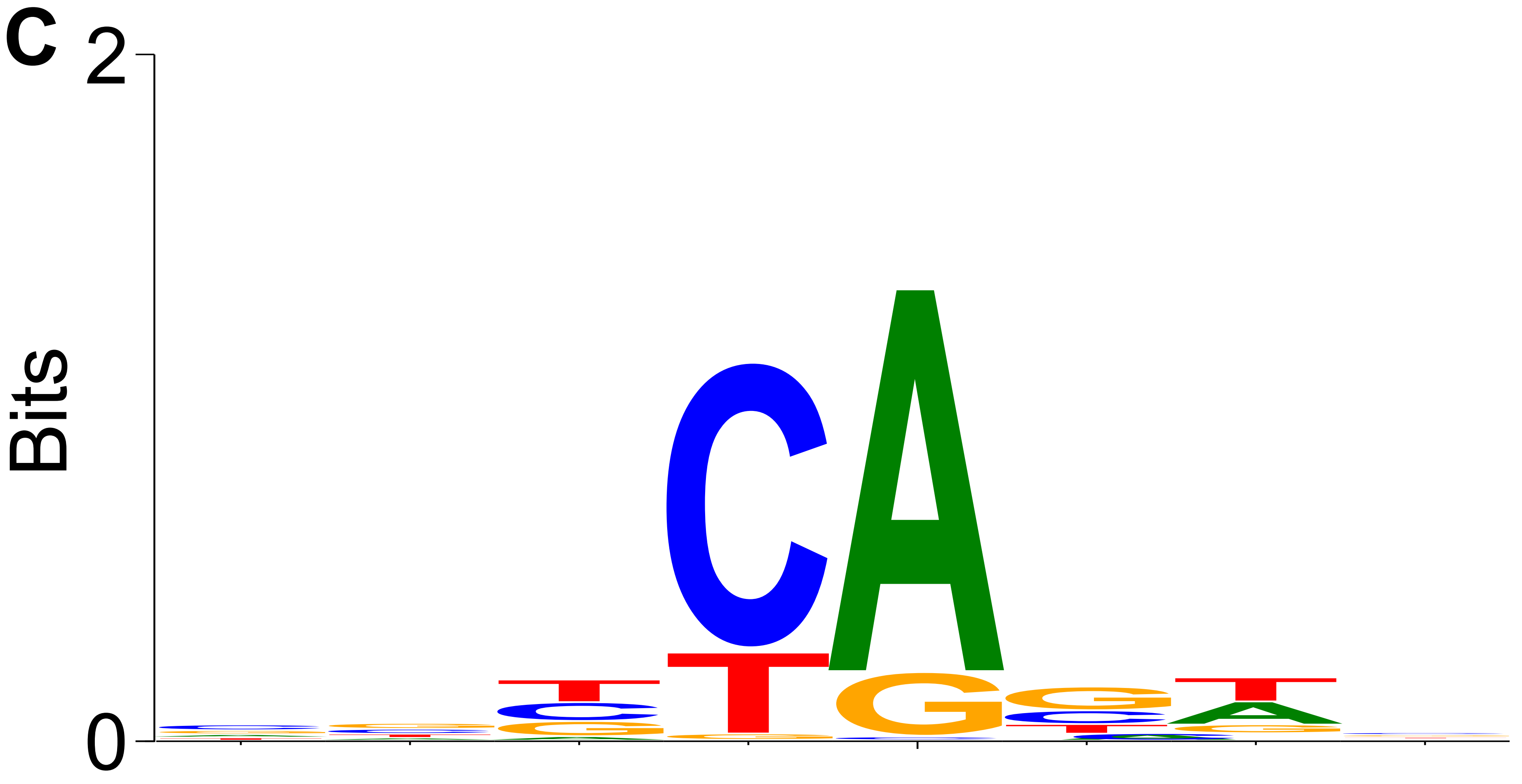

Supplementary Figure 2

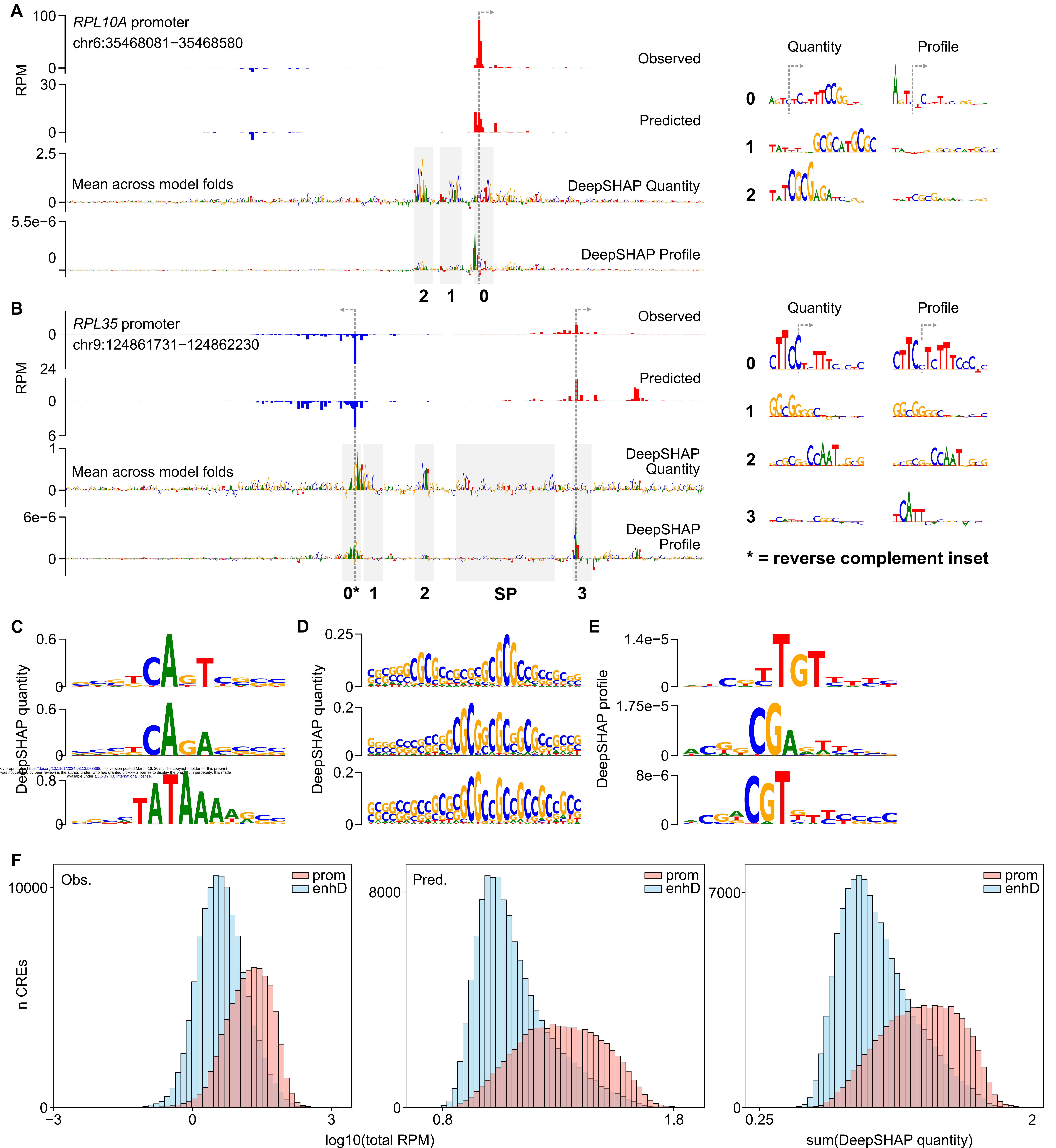

xiv preprint doi: https://doi.org/10.1101/2024.03.13.583868; this version posted March 16, 2024. The copyright holder for this preprint was not certified by peer review) is the author/funder, who has granted bioRxiv a license to display the preprint in perpetuity. It is made available under aCC-BY 4.0 International license.

Supplementary Figure 3

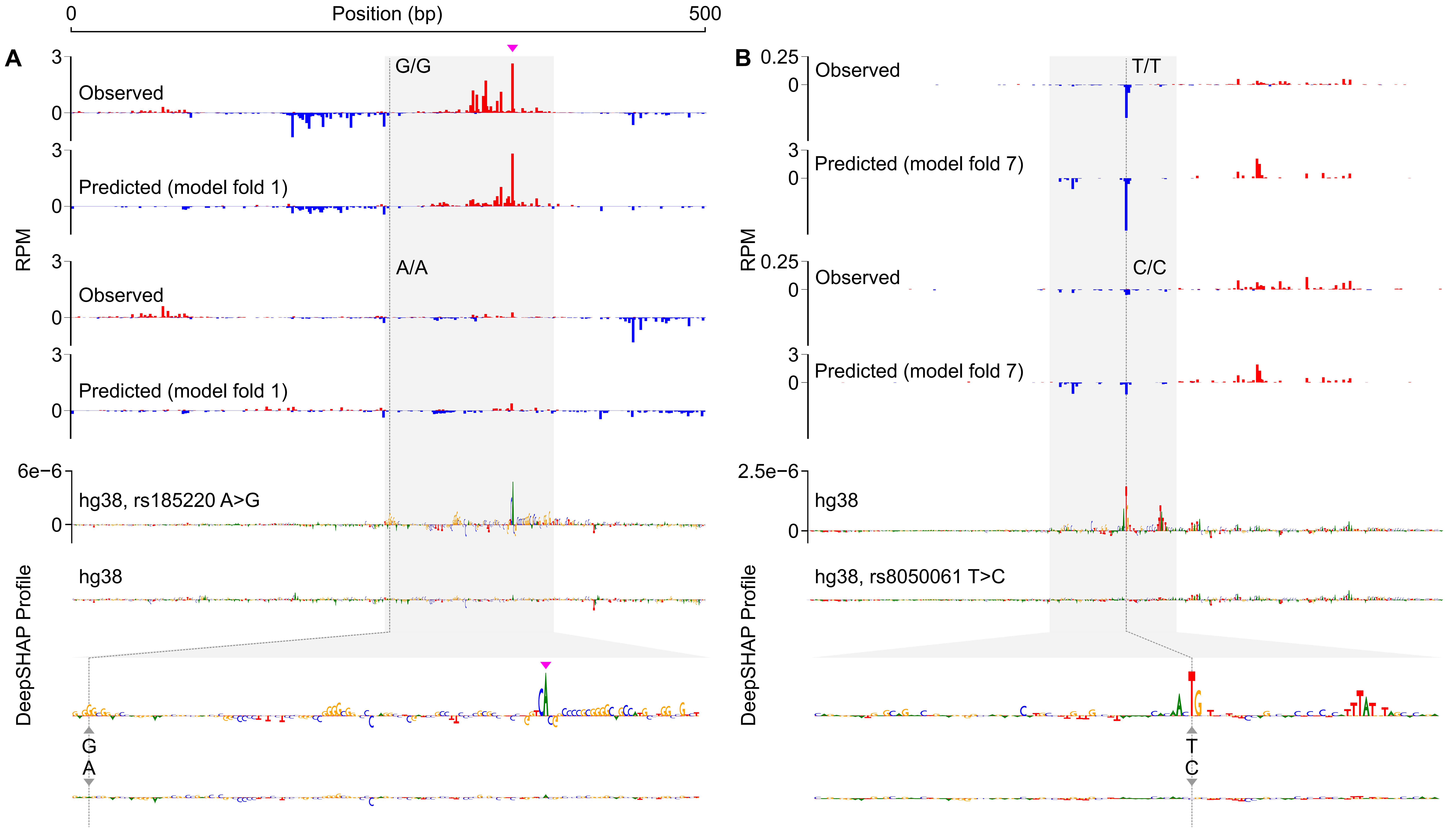

Supplementary Figure 4

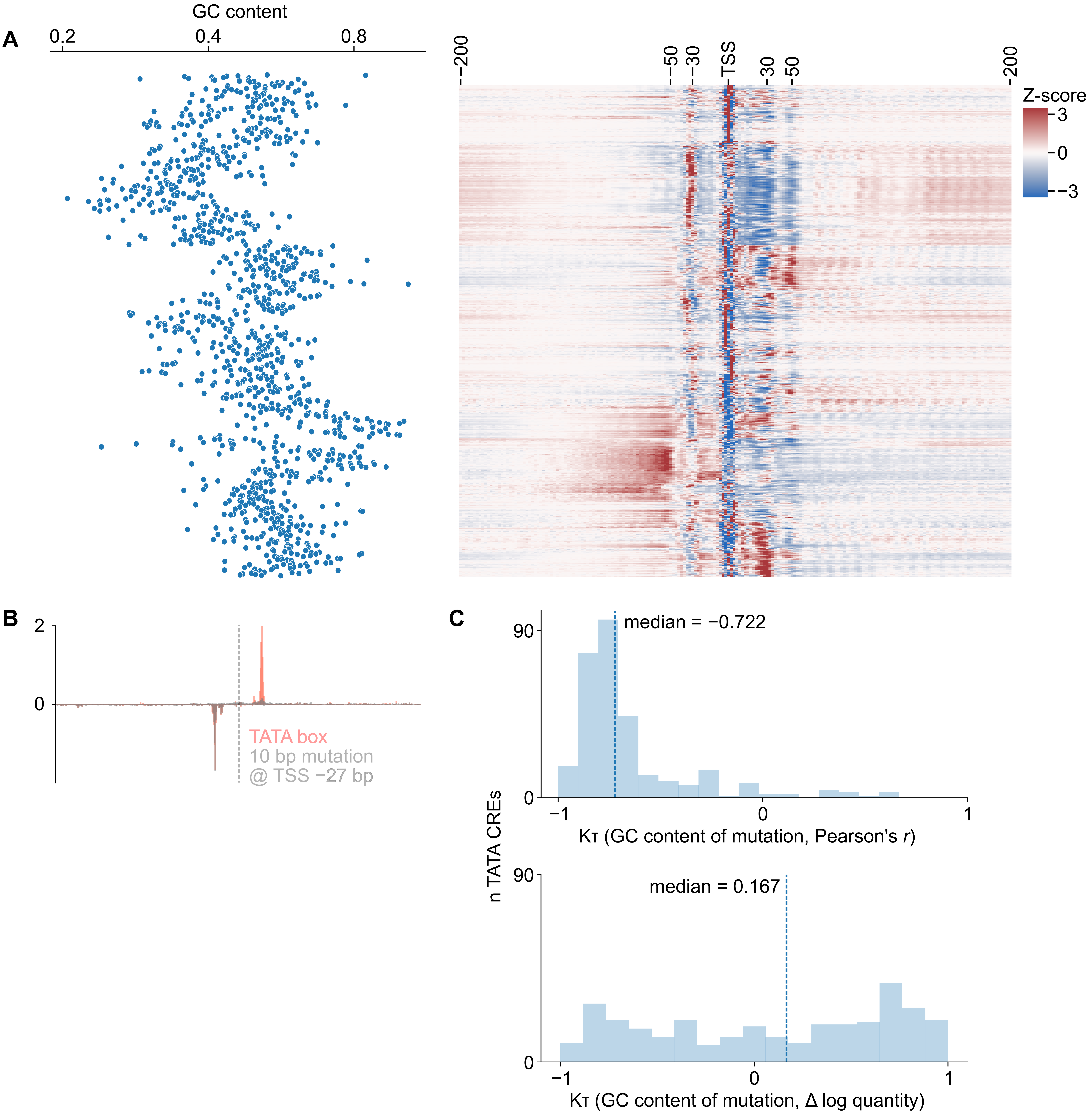

Supplement: Supplementary file 1 [file NIHPP2024.03.13.583868v2-supplement-11.pdf]
